# The Impact of Early Human Migration on Brown Adipose Tissue Evolution and Its Relevance to the Modern Obesity Pandemic

**DOI:** 10.1210/js.2018-00363

**Published:** 2018-12-18

**Authors:** Dyan Sellayah

**Affiliations:** School of Biological Sciences, University of Reading, Reading, Berkshire, United Kingdom

**Keywords:** obesity, thermogenesis, evolution, brown adipose tissue

## Abstract

Genetic factors are believed to be primarily responsible for obesity; however, an understanding of how genes for obesity have become so prevalent in modern society has proved elusive. Several theories have attempted to explain the genetic basis for obesity, but none of these appear to factor in the interethnic variation in obesity. Emerging evidence is increasingly pointing to a link between reduced basal metabolism and ineffective brown adipose tissue (BAT) thermogenesis. In fact, BAT presence and function are strongly correlated with metabolic rates and directly influence obesity susceptibility. My colleagues and I recently theorized that ancestral exposure to cold necessitated the evolution of enhanced BAT thermogenesis, which, with today’s hypercaloric and sedentary lifestyle, becomes advantageous, because thermogenesis is energetically wasteful, raising basal metabolism and burning excess calories. The opposite may be true for the descendants of heat-adapted populations. This review further reconciles global evolutionary climatic exposures with obesity demographics to understand the genetic basis for the obesity pandemic, and new insights from the most recent studies are provided, including those assessing archaic human admixture. Key genetic variants influencing BAT thermogenesis are outlined that have also been linked with climatic exposure to cold and appear to support the theory that evolutionary factors relevant to climate may have shaped the modern obesity pandemic.

Obesity has reached pandemic proportions throughout the western world and its socioeconomic impact is crippling. Obesity is particularly problematic because it is a risk factor for a plethora of metabolic diseases that each increase mortality rates. Although the relatively rapid rise in obesity prevalence over the last several decades has fueled the perception that the disease is predominantly environmental, accumulating research has strengthened the concept that genetic factors are mostly responsible for the current obesity pandemic [1]. This is not a novel idea, however. In 1986, in a study of adopted monozygotic twin pairs reared apart, Stunkard *et al.* [2] showed there was a high within-pair correlation in body mass index (BMI). Conversely, there was no relationship between BMI of adoptive parents and their adoptee. Several similar observations have been documented in comparable adoptive twin study experiments [3].

It has long been known that interindividual susceptibility to weight gain and obesity is highly variable, even under carefully controlled conditions. In a landmark paper by Bouchard *et al.* [4], pairs of adopted monozygotic twins were asked to overeat by 1000 calories/d in a carefully controlled and supervised inpatient study. The study revealed the single most important predictor of body weight gain by an individual twin was the weight gained by the other twin. In fact, there was a large variation in body weight and adiposity between twin pairs but negligible variation within twin pairs. The authors of this study concluded that heritable factors were more decisive in the promotion of obesity than were environmental factors, because twins were raised in separate environments. Energy intake and physical activity were carefully controlled for in this study; therefore, genetic factors involved in basal metabolic rate appear to be the primary determinant of obesity susceptibility in response to overfeeding.

There are three major components of daily energy expenditure: obligatory energy expenditure, which represents the energy required for upkeep of basic biochemical processes at the cellular level within the body; physical activity energy expenditure, which is the energy spent during exercise; and adaptive thermogenesis, which is the production of heat in response to environmental or dietary factors [5, 6]. Differences in adaptive thermogenesis, which raises energy expenditure beyond the obligatory energetic threshold, are potentially responsible for the interindividual variation in total daily energy expenditure and, thus, obesity susceptibility [7]. Adaptive thermogenesis involves the uncoupling of ATP synthesis from the electrochemical gradient driven by the electron transport chain. This occurs in the mitochondria of a specialist organ known as brown adipose tissue (BAT). BAT is functionally distinct from white adipose tissue, which predominates in obesity and is primarily concerned with energy storage. The energy wastage mechanism in BAT, which liberates energy in the form of heat, is mediated by uncoupling protein 1 (UCP1). The importance of BAT thermogenesis to maintenance of body temperature in small mammals was well known; however, its presence in adult humans has only recently been established [8]. Although BAT depots have been documented in newborns and shown to play a protective role in response to the negative temperature gradient between the *in utero* and neonatal environment, it was thought to deteriorate to undetectable levels by adolescence [9–11]. It was not until papers published in 2009 revealed the presence of functionally active BAT in adult humans that BAT’s potential therapeutic value in the light of the obesity pandemic was revisited [12–14].

Studies have estimated that maximally stimulated BAT can contribute as much as 20% to total daily energy expenditure and thus provide substantial resistance in the fight against obesity [15]. Another emerging concept in the field of adipose tissue research is the notion that distinct adipocytes (namely, beige or brite cells) within white adipose depots may undergo conversion to brown adipocytes under certain stimuli, such as cold exposure or sympathetic nerve stimulation [16]. This process of conversion of beige adipocytes to brown adipocytes is termed browning and is particularly therapeutically attractive in light of recent studies that suggest that under thermoneutral conditions, human BAT may be more representative of beige rather than classical BAT depots [17].

A caveat to the potential therapeutic capabilities of BAT or browning in combating obesity, however, is that BAT maybe absent or reduced in certain ethnic groups. It remains to be determined whether recruitment of BAT or browning mechanisms is equally limited in these ethnic groups, and additional research is required to answer this important question. The question of why various populations have reduced BAT functionality compared with others is an intriguing one, and answers to this immensely important mystery may be found in the evolutionary history of the ancestors of modern humans.

## 1. Evolution of BAT

Nonshivering thermogenesis (NST) is a form of adaptive thermogenesis that facilitates rewarming from torpor or hibernation and/or maintains homoeothermic endothermy, which is defined as the ability to raise and maintain core body temperature above and beyond that derived from the external environment [18]. The evolutionary origins of NST, which is mediated by BAT, remain controversial, however. Classical BAT depots have thus far only been discovered in mammals, suggesting that the tissue may have evolved to fuel the endothermic requirements of early mammals [19]. *UCP1* predates the origins of mammals, however; it has been found in teleost fish [20]. Because fish do not possess BAT and are generally not endothermic, a nonthermogenic role for ancient UCP1 has been postulated; however, it is possible this gene may have provided a localized thermogenic role, such as in cranial endothermy in specialized heater cells within the brains of certain fish species [21]. In line with this argument, cold exposure results in increased brain UCP1 expression in the common carp [22], though more direct evidence for thermogenic properties of UCP1 in fish is required.

UCP1-dependent NST in BAT has been observed in various eutherian mammals but was thought to be absent in most marsupials [20]. Molecular phylogenetic analysis suggests that thermogenic properties of UCP1 evolved in eutherian mammals in the latter half of the Cretaceous Period [23]; however, a study has shown that a UCP1 homolog is expressed in BAT of some marsupials [24], albeit not in adults. Other studies have shown that marsupial species possess BAT but do not rely on it for cold resistance [25]. Regardless of its origins, UCP1-dependent NST in eutherian mammals appears to have promoted higher metabolic rates and allowed for a stable body temperature, independent of the ambient external environment, enabling higher rates of reproduction and facilitating rapid adaptation to a range of environmental niches. Eutherian mammals, with the evolution of UCP1-dependent BAT thermogenesis, were able to hunt and forage at dusk and during the night when predatory reptiles were inactive. Owing to BAT evolution, eutherians were freed to inhabit a greater diversity of environments and establish themselves in previously uninhabitable climates. As such, the evolution of endothermy is believed to have fueled eutherian mammalian radiation at the end of the Cretaceous Period, wherein >4000 species of eutherians appeared on the fossil record almost simultaneously. Findings of a recent study that showed greater species diversification in *UCP1*-possessing taxa compared with *UCP1*-absent taxa, appear to support this hypothesis [26].

Eutherian mammals diverged from the lineage with marsupials some 150 million years ago [27–29]. Approximately 60 million years ago, primates emerged in eastern Africa. Approximately 4 million years ago, an early hominid, *Australopithecus afarensis,* evolved into *Homo erectus*, whose descendants include all archaic human species, such as *H. neanderthalensis* (Neanderthals), *H. denisova* (Denisovans), and modern humans [30].

Although eutherian mammals diversified and adapted to a range of ecological niches ranging from tropical to polar, by the time modern humans evolved, ∼2 million years ago, they would have been highly adapted to the intense heat and arid conditions of sub-Saharan Africa [31]. The requirement for heat resistance over cold tolerance would have reduced the evolutionary necessity for UCP1-dependent NST in BAT. As archaic and modern humans migrated out of Africa and began to inhabit northerly latitudes, however, the presence of UCP1-dependent NST would have been a selective advantage that provided sufficient acclimation to cold. Thus, evolutionary exposure to cold may have influenced the selection for genes involved in NST in BAT. To this end, a genetic variant in the b-3 adrenergic receptor (*ADRB3*) gene has been found in such high frequencies in all nonhuman primates that it has reached fixation [32]. ADRB3 on the surface of BAT stimulates lipolysis and activates NST [18]. The variant found in nonhuman primates is thrifty: It leads to the reduction in lipolysis and blunted NST [33]. Intriguingly, an energy wastage variant of ADRB3 that promotes lipolysis and NST has been found only in humans, supporting the notion that the common ancestor of nonhuman primates and hominids who lived in Africa had not required energy wastage alleles that augmented thermogenesis [32].

## 2. Out of Africa: Cold Adaptation and Evolution of BAT

Various conflicting theories of modern human dispersal out of Africa have muddied the waters on a precise timeline of events. The most viable theory, based on genetic evidence, suggests that all contemporary non-Africans are descended from a small population of migrants that left Africa ~60,000 years ago [34, 35]. Although earlier migration events were likely, they were not ultimately successful; there is very little trace of genetic material from an earlier migration event in present populations. The caveat remains, however, that only a small fraction of the global non-African population has thus far been genetically tested and it is thus possible that traces of genetic material from an earlier migration event will be discovered. Before this decisive out of Africa migration of modern humans, *H. erectus* successfully migrated off of the continent, encountering cold climates for the first time. Approximately 500,000 years ago in Eurasia, Neanderthals evolved from the *H. erectus*. These short-statured species were highly adapted to cold environments and likely possessed high basal metabolic rates and elevated rates of vasoconstriction for heat preservation [36–38]. Another archaic species, the enigmatic Denisovans, whose paleontological remains were recently excavated in Siberia, perhaps represent the far-eastern extent of the Eurasian Neanderthals, sharing anatomical features and being equally adapted to extreme cold [39]. Although archaic humans were long thought to have not interbred with modern humans, it has emerged that all non-African modern humans share genetic material with these archaic species, equating to between 1% and 6% of the genome [40]. Given a lack of evidence of admixture of archaic DNA in the genomes of contemporary Africans, it has been concluded that the admixture arose sometime after the out of Africa migration event of modern humans 60,000 years ago somewhere in Eurasia [41]. Because archaic species had adapted to cold climates for hundreds of thousands of years, before modern humans arrived, it would be reasonable to speculate that archaic species bestowed cold-adaptive genes (*e.g.*, those involved in BAT NST) during admixture, which proved to be a selective advantage and facilitated rapid adaptation to a sudden change of environment.

**Figure 1.**
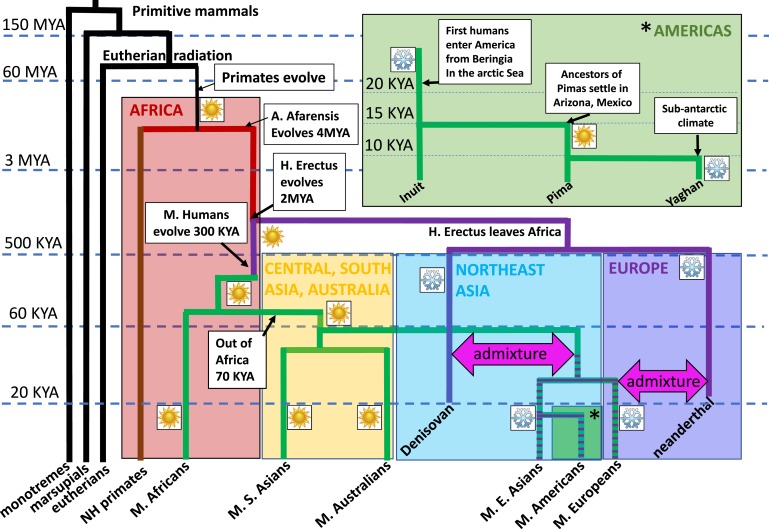
Diagrammatic representation of the concept of human adaptation to climate and the selection for BAT genes. Eutherian mammals evolved 150 MYA. Approximately 65 to 70 MYA, BAT fueled mammalian radiation, in which most orders of mammals alive today evolved. Primates evolved in Africa, and hominids diverged from the line with nonhuman primates, giving rise to *Australopithecus aferensis* ~4 MYA. *Homo erectus* speciated from *Australopithecus* ~2 MYA. Between 500 and 400 KYA, *H. erectus* left Africa and adapted to cold climates (snowflake icon) for the first time. The species later diverged into two archaic human species differing in their geographic distribution. Denisovans lived in northeast Asia; Neanderthals lived in Europe and west Asia. By the time the ancestors of all non-African modern human populations left Africa 70 KYA, they were highly adapted to heat (sun icon); however, as they reached more northerly latitudes in northeast Asia, genes for cold adaptation would have been advantageous. In addition to selective pressures acting on modern human DNA, archaic admixture and subsequent adaptive introgression of cold-adaptive archaic genes may have aided modern human survival in cold climates. The story is rather more complex in the Americas where, within a few thousand years, early migrants in America encountered subarctic conditions in Beringia to tropical conditions in Arizona and Mexico (ancestors of Pima Indians) to sub-Antarctic conditions in Tierra del Fuego (ancestors of indigenous Yaghan). Descendants of cold-adapted populations would likely have evolved efficient BAT capacity, whereas the opposite may be true of descendants of heat-adapted populations. Such evolutionary factors may explain why the descendants of heat-adapted populations, such as modern Africans, south Asians, and Pimas, have reduced metabolic rates and greater obesity susceptibility compared with cold-adapted populations such as modern east Asians and Europeans. E, east; KYA, thousand years ago; M, modern; MYA, million years ago; NH, nonhuman; S, south.

There appears to be little evidence for advantageous archaic human alleles in contemporary modern humans, however. In fact, recent experimental evidence points to purifying selection wherein deleterious archaic human alleles have been systematically purged by selection [42]. Nevertheless, certain genetic loci that presumably conferred survival advantages appear to have been under positive selection, most notably in genes involved in immune responses [43]. Moreover, adaptive introgression of archaic genes for climate adaption in modern humans has been observed. For instance, Denisovan gene variants enabled early modern human inhabitants of Tibet to adapt to the extremely cold and hypoxic high-altitude environment [44]. Intriguingly, another study suggests that genes involved in BAT thermogenesis in Greenland Inuits and presumed to support genetic adaptation to cold may have been adaptively introgressed from archaic genomes. Racimo *et al.* [45] analyzed the genomes of contemporary Inuit populations and found that a high-frequency allele in Inuits that corresponds to the T-box transcription factor 15/mitochondrial tryptophan tRNA synthetase 2 (*TBX15/WARS2*) locus. The authors found that this gene was likely the result of introgression from archaic populations, most likely the Denisovans. Interestingly, the *TBX15/WARS2* locus has also been identified in genome-wide association studies (GWAS) as a being associated with BMI in Europeans [46]. This locus affects adipogenic differentiation and metabolism of brown and beige adipocytes and thus potentially mediates thermogenic responses in NST [47]. The introgressed allele, which is postulated to provide selective advantages in terms of adaptation to cold environments, was found at higher frequencies in contemporary east Asian populations than in contemporary Europeans, and at lowest frequencies in contemporary Africans [45]. A similar observation was made in a separate study in which an archaic leptin receptor allele, presumed to be associated with increased thermogenesis, occurred at higher frequencies in contemporary east Asian populations than in Europeans; both groups descend from cold-adapted early humans [48]. The latter study did not detect the *TBX15/WARS2* signal, though it did not include genetic analysis of contemporary populations living in extreme cold, such as Inuits. The differential allele frequency for certain cold-adaptive variants in equally cold-tolerant populations suggests that cold adaptation may have evolved independently in Europeans and east Asians and may be related to the proportion of archaic admixture (east Asian populations have a higher percentage admixture than Europeans). Neanderthal ancestry has increased the expression of genes that promote lipid catabolism in Europeans vs Asians [49]. Given that NST in BAT is activated by the release of fatty acids from lipolysis, enhanced lipid catabolism may have conferred resistance to cold [32]. Similar to differential mechanisms of cold adaptation, reduced skin pigmentation in Europeans and east Asians appears to be conferred by distinct genes, suggesting convergent evolution in similar traits that are adaptive to northerly latitudes [50]. The timing and frequency of admixture between archaic and modern humans is currently the subject of debate, though recent estimates suggest numerous admixture events have been more frequent in east Asian populations, potentially explaining the higher proportion of archaic DNA in this population [40, 51]. It also appears that later waves of Neanderthals interbred with the ancestors of modern east Asians after the former had encountered and admixed with the ancestors of modern Europeans [40]. Figure 1 outlines potential archaic/modern human admixture events that may have facilitated early modern human inhabitants of northeast Asia and Europe to survive in extreme cold.

Whether archaic admixture has influenced the presence of cold adaptive genes and thereby influenced BAT presence and function in modern humans remains to be confirmed. What is becoming increasingly clear, however, is that contemporary modern human populations display considerable variation in functional BAT, which may be related to latitude. Thus, presence of functional BAT in contemporary populations may have been influenced by evolutionary factors driven by ancestral exposure to cold environments. Certainly, for a single gene or allele to strongly influence adiposity and cold adaption simultaneously, BAT thermogenesis must be a leading candidate in their combined causality. Genetic variants that have a strong association with BMI and latitude and that have been shown to function in BAT/NST are highlighted in Table 1.

**Table 1.** Genetic Variants Associated With Latitude, Obesity, and BAT Thermogenesis

Gene/Locus	Function	Finding	First Author
UCP1	Thermogenesis	Haplotype found at higher frequencies in Europeans vs Africans	Nishimura [53]
PRDM16	Brown adipogenesis SERCA uncoupler	SNPs associated with latitude, believed to function in cold adaptation	Quagliariello [54]
THADA	Lipolysis	Variants strongly correlate with BMI and latitude	Moraru [57]
ADRB3	Thermogenesis	Energy-storage variant higher in Africans and Pima Indians	Walston [33]
TBX15/Wars2	Brown adipogenesis	Archaic allele associated with cold adaptation and BMI	Racimo [45]
TRIB2	Brown adipogenesis	Obesity-resistant variant associated with cold adaptation in east Asians	Nakayama [77]

A study revealed that BAT volume and NST were reduced in south Asians, which had a negative impact on basal metabolism and energy expenditure [52]. Nishimura *et al.* [53] have shown that a *UCP1* haplotype consisting of a series of single nucleotide polymorphisms (SNPs) in the UCP1 locus increased NST and were associated with increased basal metabolism. Furthermore, the *UCP1* haplotype was found at higher frequencies in European populations (63%) compared with Africans (6.5%). This study supports the notion that genes for enhanced NST in BAT may have undergone positive selection as modern humans migrated out of Africa and inhabited colder regions.

Another thermogenic gene in which allelic variants exhibit a latitudinal pattern of expression is PR/SET domain 16 (*PRDM16*), a master regulator of brown and beige adipogenesis. A study has revealed that SNPs in *PRDM16* were differentially expressed in modern European populations and that the SNPs were found at higher frequencies in northern European populations than in southern European populations [54]. Such latitudinal-based genetic diversity in Europeans is observed in other loci and reflects the known demarcation in the genetic landscape between northern and southern Europeans, potentially driven by climatic selection pressures [55].

Another gene that is differentially expressed on the basis of global distribution and climatic factors is the thyroid adenoma-associated (*THADA*) gene. SNPs in the *THADA* gene have experienced one of the highest rates of positive selection in modern humans [56]. These mutations, which have archaic origins, have a heavily latitudinal basis, being found at higher frequencies in northerly latitudes and colder climates, suggesting a role in cold adaptation. In support of this notion, Moraru *et al.* [57] found that THADA binds the sarco/ER Ca^2+^ (SERCA), which augments NST by providing a UCP1-independent means of uncoupled respiration in BAT of rodents [58]. *THADA* mutant flies lacking in SERCA activity were cold sensitive and prone to obesity. Thus, THADA may play a UCP1-independent role in thermogenesis and cold adaptation and may thus influence ethnic variations in obesity susceptibility.

A mutation in the *ADRB3* gene that favors energy storage, because of reductions in lipolysis and NST, which suppress basal metabolism, occurs at higher frequencies in people of African descent compared with those of European descent [33]. The energy wastage version of the allele (trp64) has been found only in humans and may support cold adaptation [32]. Interestingly, the energy storage variant of *ADRB3* has the highest frequencies in Pima Indians (more so than Africans), who have evolved to live in the extreme heat of Arizona. Of note, Pima Indians, who also have some of the highest rates of obesity in the world, have been cited as evidence in support of the thrifty gene hypothesis, which suggests that genes favoring energy storage during times of famine have become maladaptive in current Western society [59]. It is more plausible, however, that climatic exposure had more of a bearing on selection for energy wastage vs energy storage as opposed to genes concerned solely with energy conservation.

## 3. Energy Expenditure Variations by Ethnicity

Given that ancestral exposure to cold influenced genes for functional BAT and NST, and that BAT function varies greatly between individuals, it is plausible that ethnicity, which represents a direct genetic link to ancient ancestry and climatic positive selection, reflects BAT function and thus predicts basal metabolic rates. Evidence in favor of this concept has been mounting in recent years [60] and suggests that ethnic groups whose ancestry lies in Africa, south Asia, and other regions wherein extreme cold exposure has been largely absent have reduced BAT activity and lower basal metabolic rates. Numerous studies have shown that basal metabolic rates are lower in individuals of African ancestry compared with those of European ancestry, which predisposes the former group to obesity and may inhibit weight loss [61]. This lowered metabolism has been linked to reduced NST and BAT activity [62]. A study has shown that even after adjusting for lean mass (muscle is more metabolically active), age, sex, and other potential confounders, African ancestry is associated with reduced basal metabolism and lower lipid oxidation compared with European ancestry [63]. Interestingly, this reduced basal metabolism has been identified in young African American women of normal weight and with no history of weight problems, suggesting a causal relationship in obesity [64]. Interestingly, the presence and degree of European ancestry in African individuals were positively correlated with basal metabolism, confirming that African ancestry is associated with reduced metabolism, whereas European ancestry is associated with higher metabolic rates [65]. Alarmingly, complete African ancestry without European admixture lowers basal metabolism by as much as 160 kcal/d. This is not an insignificant amount and equates to a >7-kg weight gain per year.

Taken together, the findings of these studies strongly suggest that reduced basal metabolism and obesity susceptibility in Africans may be due to insufficient BAT function. African Americans have consistently higher obesity rates across the United States and other Western countries compared with whites [66]. It stands to reason that the lack of ancestral exposure to cold is the predominant factor in a blunted BAT activity observed in this ethnic group. Similar to Africans, south Asian ancestry is associated with decreased BAT volume and function [52]. South Asians living in Western countries have greater susceptibility to obesity and metabolic disorders such as diabetes and cardiovascular disease, which maybe mediated by lowered BAT activity.

On the other end of the spectrum, east Asian ancestry (*e.g.*, Chinese, Japanese, Korean) is associated with relative obesity resistance [67–69]. Individuals of Chinese ancestry in industrialized countries have some of the lowest rates of obesity despite an increasingly Western lifestyle. Certainly, of the ethnic groups in the United States, east Asians are the least likely to be obese. Interestingly, the ancestors of east Asia who lived in Siberia for thousands of years were highly adapted to extreme cold [60]. In fact, the unique shape of the skull, particularly the angles of the cheekbones of east Asians, is widely believed to protect from extreme cold [70]. Various indigenous peoples of Tibet and Siberia who are genetically similar to contemporary Chinese and Japanese peoples share cold-adaptive traits and have significantly elevated basal metabolic rates [71, 72]. This pattern of higher basal metabolism in populations whose ancestors were highly cold adapted has been robustly replicated in various studies [73–75]. It is noteworthy that an adaptive variant of the tribbles pseudokinase 2 (TRIB2) gene, which was found to have undergone positive selection during the last glacial maximum, was also associated with enhanced expression of thermogenic gene expression in Japanese populations. Moreover, the adaptive variant is protective against obesity, presumably through elevated levels of thermogenesis [76, 77]. Thus, TRIB2 represents a gene that has demonstrable associations with climate adaptation and adiposity potentially through thermogenic activity.

Archaic human admixture from Neanderthals and Denisovans likely contributed to enhanced cold adaption in east Asians and may explain why these individuals exhibit some of the highest rates of archaic admixture [78]. More studies are required, however, to determine whether elevated basal metabolism in indigenous peoples of the arctic and subarctic is due to high rates of BAT NST.

To explain the genetic basis for the obesity pandemic in industrialized countries, various conflicting theories have been presented. The thrifty gene posits that since the advent of farming, modern human populations have placed their food security at the mercy of unpredictable climatic environments, which necessitated the selection for genes that enabled effective energy storage during times of famine [79, 80].

## 4. Drifty vs Thrifty Gene Hypotheses

A credible alternative explanation to the thrifty gene hypothesis was presented by John Speakman, who argues that, contrary to undergoing positive selection, genes that promoted fat storage were the result of genetic drift [81, 82]. When humans were able to band together and coordinate their foraging, hunting, and social activities, as well as evade prey by innovative tactics such as the use of fire, they were able to effectively remove themselves from danger without relying on genes for leanness, physical strength, and athleticism. Speakman argues that this “predation release” led neither to purifying selection nor positive selection for thrifty alleles. The thrifty alleles were simply neutral and allowed to drift, explaining their high prevalence in today’s modern human gene pool.

Work from Speakman’s group has revealed very little evidence for positive selection for thrifty genes. Only four of nine positive selection signals for BMI genes previously detected in GWAS were in genes linked to energy and fat storage [83]. In fact, for the majority of BMI-associated variants (five of nine), positive selection favored the protective allele (*i.e.*, they favored leanness). Moreover, three of the four thrifty alleles that underwent positive selection had selection signals before 30,000 years ago, many decades before the advent of farming. This lack of strong selection for thrifty alleles, evidence for the positive selection for protective alleles, and the timing of selection for thrifty alleles are strong evidence against the thrifty gene concept.

## 5. The Question of Obesity Demographics

Another flaw in the thrifty hypothesis is its inability to reconcile evolutionary genetics with the contemporary obesity demographics. Obesity rates in Western countries such as the United States and the United Kingdom are not equal across ethnicities, as previously highlighted [60]. Studies have consistently shown that African ancestry is associated with the highest obesity rates, followed by south Asians. East Asians and whites have the lowest rates of obesity. Although cultural and socioeconomic factors undoubtedly play a role, increasing evidence for genetic contributions to this observation cannot be refuted. The thrifty gene hypothesis is unable to explain why such interethnic variation in obesity has manifest in recent times. Given the relative obesity resistance in east Asian ancestry (particularly those with Chinese and Japanese ancestry), the theory implies there has been relatively little famine observed in Chinese and Japanese history. Quite the contrary, however: Some of the most catastrophic famine episodes have been reported in east Asia within the last 1000 years [84]. Gluckman and Hanson [85, 86] have argued that very little evidence exists for European famines on a scale sufficient enough to cause large-scale changes in allele frequency. Moreover, statistical analysis has revealed that food security has been surprisingly consistent throughout evolutionary history, regardless of agricultural, foraging, or hunting practices [87].

## 6. Toward a Unifying Theory

Although it may be argued that the ethnic variation in obesity may represent genetic drift due to founder effects in populations such Samoans and Pima Indians, the drifty genotype hypothesis cannot effectively explain why various ethnic groups such as east Asians are protected from obesity. An alternative explanation for the modern obesity pandemic might be related to the selection (or not) for energy expense alleles related to NST in BAT. This viewpoint represents a form of maladaptive scenario in which genes that provided heat adaption (*i.e.*, lack of BAT NST) rather than cold adaptation become disadvantageous in the modern high-calorie, low physical activity society. The positive selection for BAT genes on the other hand, which historically provided effective NST to deal with cold, also conferred higher metabolic rates. This is metabolically advantageous in today’s society because, in addition to conferring higher basal metabolic rates, BAT NST can be stimulated by excess fat and caloric intake, thus providing a way to burn off excess energy intake. This may explain why east Asians such as Chinese and Japanese living in the United States are obesity resistant, despite consuming similar nutrition and having similar a lifestyle [60].

An interesting nuance in obesity demographics is the disparity in obesity susceptibility among mongoloids, particularly native Americans (including Pima Indians), Pacific Islanders, and east Asians, who share a high degree of genetic similarity and are descended from a common ancestor [88]. Interestingly, although all three groups have cold-adapted ancestry, only the east Asians appear to have high metabolic rates that confer obesity resistance [89, 90]. When the ancestors of native Americans, including Pima Indians, entered the Bering land bridge during the last ice age, much of northern North America was covered in ice. Given evidence of an extended presence in Bering before entering the Americas ~12,000 to 13,000 years ago and a population bottleneck, these people would have been highly cold adapted [35, 91]. As the first American settlers traveled south along the coast toward Arizona and Mexico where Pima Indians settled, they would have had to reacquire genes for heat adaptation [92]. In fact, Native Americans exhibit physiological responses to salt loss during sweating that are comparable to those of Africans, suggesting that cold adapted mongoloids who entered the Americas and migrated rapidly southward were heat adapted by the time they settled in arid Arizona and Central America. This hypothetical purging of cold adaptation genes may explain the lower metabolism and susceptibility to obesity in Pima Indians [92]. Native Americans who remained in circumpolar regions, such as Inuits, remain cold adapted and have higher metabolic rates. Interestingly, a subpopulation of the ancestors of Pimas continued their rapid southerly migration from central America, reaching the southern tip by 10,000 years ago. Their descendants, the indigenous Yaghan people, appear to have high metabolic rates and are highly adapted to cold [93]. Thus, evolutionary climatic exposures that influenced NST and BAT function may explain the ethnic variation in basal metabolism and obesity rates today. This concept is illustrated in Figure 1.

Another factor that may affect the therapeutic potential of browning capacity and BAT thermogenic function is that most people who are obese live in industrialized countries with central heating and are not generally under cold stress. Given that studies have shown that chronic cold stress or sympathetic nervous stimulation is required to maintain the oxidative and thermogenic properties of beige cells [94], it remains to be fully determined what long-term effects artificial heating have had on browning capacity in humans, though studies in rodents indicate that whitening of beige cells in response to thermoneutral conditions may be reversed by cold acclimation [95].

More evidence is required to directly assess the impact of evolutionary climatic selection pressures on genes for BAT function across ethnic groups. At present, the vast majority of GWAS on BMI have been conducted in Europeans. A further limitation to the GWAS approach is understanding the functional relationship of the SNP to metabolic function and to determine the precise causal nature in obesity susceptibility. The case of the *FTO* gene is prime example of the inherent difficulties presented by GWAS in providing functional context [96]. Nevertheless, although GWAS have identified common SNPs for BMI across ethnicities, the vast majority appear to be race or ethnicity specific, which appears to support the view that ethnic variations in obesity susceptibility should be considered when discussing the evolutionary origins of obesity [97].

Whatever the cause, considerable evidence points to the fact that certain ethnic groups have lowered basal metabolism and thus daily caloric requirements vary greatly by ethnicity. Given that a large proportion of dieters in the United States and Europe use calorie-counting programs [98], it is incumbent on national health services such as the National Health Service in the United Kingdom and the National Institutes of Health in the United States to issue ethnicity- and race-specific guidelines on caloric requirements to prevent overconsumption. In recent years, race-specific guidelines for clinical cardiovascular measures such as high-density lipoprotein and low-density lipoprotein cholesterol have been issued for south Asians [99]. Such targeted strategies are seen as a positive step in tackling the ethnic variation in heart disease, but similar approaches should be considered for guidelines on caloric consumption.

In summary, the genetic cause of the modern obesity pandemic is highly complex and more questions than answers remain. What is clear, however, is that obesity rates vary greatly by ethnicity and this is reflected in basal metabolic rates. The genetics associated with interethnic differences in basal metabolic rates may lie in our evolutionary past. Specifically, the selection for genes associated with NST in BAT, which influence metabolism, may have been shaped by differential climatic exposures since the migration out of Africa of modern humans ~60,000 years ago.

## Abbreviations:

ADRB3: b-3 adrenergic receptor
BAT: brown adipose tissue
BMI: body mass index
GWAS: genome-wide association studies
NST: nonshivering thermogenesis
SNP: single nucleotide polymorphism
TBX15/WARS2: T-box transcription factor 15/mitochondrial tryptophan tRNA synthetase 2
THADA: thyroid adenoma-associated
UCP1: uncoupling protein 1
